# Acute-onset stomatitis in a young European woman visiting Brazil: a case report

**DOI:** 10.3389/froh.2025.1630797

**Published:** 2025-07-16

**Authors:** Aleksandra Kierczak, Tomasz Smiatacz, Marta Katkowska, Katarzyna Garbacz

**Affiliations:** ^1^ Oral Microbiology Student Scientific Club, Medical Faculty, Medical University of Gdansk, Gdansk, Poland; ^2^ Department of Infectious Diseases, Medical Faculty, Medical University of Gdansk, Gdansk, Poland; ^3^ Department of Oral Microbiology, Medical Faculty, Medical University of Gdansk, Gdansk, Poland

**Keywords:** stomatitis (mucositis), herpangina, HSV-1, enteroviruses (EVs), travel

## Abstract

We report a case of acute, severe stomatitis in a 20-year-old European woman shortly after her arrival in Brazil following participation in a multi-day carnival. The clinical picture was consistent with either primary herpes simplex virus type 1 (HSV-1) infection or herpangina caused by enteroviruses. Due to the absence of virological testing, a definitive diagnosis was not established. Empirical treatment with corticosteroids and antibiotics was initiated, while antiviral therapy was omitted. The patient's symptoms progressed significantly before resolving with supportive care within ten days. This case underscores the diagnostic challenges of viral oral infections in adults, especially in travelers exposed to novel pathogens. It highlights the importance of bedside diagnostic tools, the potential risks associated with empirical corticosteroid use, and the need to enhance clinician awareness of viral stomatitis as a significant and potentially debilitating condition in adult patients.

## Introduction

Stomatitis represents a clinically significant problem in adults and is often caused by viruses. While many cases are self-limiting, certain infections can lead to serious complications, hospitalization, and long-term management strategies. The severity and impact of stomatitis vary depending on the causative virus, the patient's immune status, and access to healthcare. Atypical symptoms (e.g., no vesicles, localization in only one place) of infection can make diagnosis difficult. Viral stomatitis/gingivostomatitis may be confused with tonsillitis, canker sores, candidiasis, and allergic reactions, which can be a letter to inappropriate therapies. In addition, the lack of standard use of antigenic or PCR tests and routine diagnosis based solely on the clinical picture can lead to misdiagnosis and delayed treatment. Viral stomatitis in adults is clinically significant due to its high prevalence, impact on quality of life, recurrence, and risk of complications. Strengthening public health awareness, early diagnosis, and preventive measures is key to reducing the burden of these infections (1, 2).

In this paper, we report a case of severe stomatitis in a 20-year-old female student, during her second week in Brazil following a four-day carnival.

## Case report

**Figure 1:**
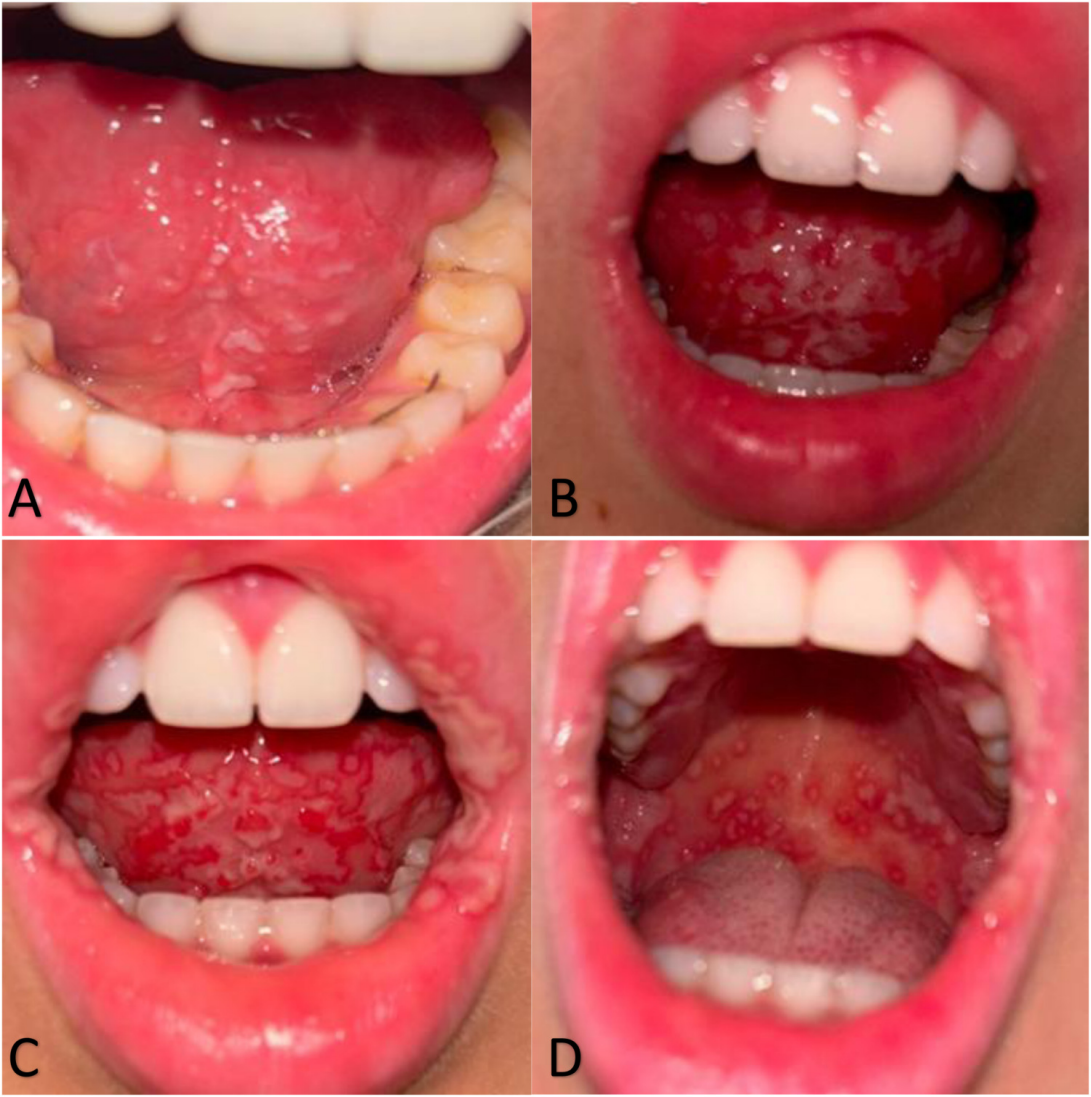
Progression of oral mucosal lesions over the first three days of illness. **(A)** Day 1: White, unremovable changes/residue under the tongue. **(B)** Day 2: Progression of white plaque-like changes spreading beneath the tongue and extending to the lips. **(C,D)** Day 3: Severe extension of erosive lesions across the oral cavity, including the sublingual area and inner cheeks.

In February 2024, a 20-year-old female student from Poland, during her second week in Brazil, was admitted to the Emergency Department in Santa Casa de Misericórdia de Barretos following a four-day carnival. Two days after carnival, the patient reported whitish lesions underneath the tongue, accompanied by significantly swollen and tender cervical and submandibular lymph nodes (Figure 1A). The patient additionally complained about headache, light sore throat, sub-febrile state, loss of appetite, lethargy, and acute neck stiffness due to severe lymphadenopathy. The first diagnosis made was candidiasis, and fuconazole was prescribed as empirical treatment. The patient was sent back home.

On the second day, her symptoms significantly worsened, and she developed progressive trismus, decreased oral intake, drooling, worsened bilateral neck pain, and hot potato voice. Moreover, new symptoms appeared, including severe fever reaching 38.2°C which did not respond to medication, and white erosions spreading significantly more underneath the tongue (Figure 1B). On the same day, the patient was referred to both infectious disease and otolaryngology specialists, who collectively diagnosed a viral infection likely caused by Coxsackievirus A, suggestively herpangina, alongside a mixed bacterial infection. The previous diagnosis of oral candidiasis was no longer considered.

On the third day of symptomatic treatment, severe and acute erosions appeared (Figure 1C). The patient started complaining about significantly worsening pain in the oral cavity and throat when swallowing, hence refusing to eat and drink. High fevers reaching 38.7°C were still developing for another 3 days, during which she refused to eat any solid food. She was fed with only liquids and water in low amounts. She described the sixth day of symptoms as the worst in case of pain, general sensation, and oral lesions severity. Erosions developed into severe oral ulcers, further spreading outside the mouth and appearing inside the cheeks, on the soft palate, gums, pharyngeal arch and lips, and outside of the oral cavity (Figure 1D).

On physical examination, the patient appeared visibly ill and reported severe pain in the oral cavity. The cervical and submandibular lymph nodes were bilaterally enlarged, firm, tender, and reached approximately the size of plums. There was a marked trismus, with a maximal mouth opening of only about 20 mm, making it difficult for the patient to speak or eat. She was only able to utter short phrases due to pain during mouth opening and swallowing. Examination of the oral cavity revealed extensive ulcerations involving the underside of the tongue, buccal mucosa, soft palate, gums, lips, and pharyngeal arches. The lesions were painful, deep, and associated with significant salivation and odynophagia. The patient reported a complete inability to consume solid foods for approximately 4 days and was sustained on liquids only, resulting in an estimated 5 kg weight loss. Despite the severity of local symptoms, there were no signs of systemic instability.

On the sixth day of illness, a complete blood count (CBC) was performed. The results revealed that the red blood cell count, hemoglobin, hematocrit, and platelet levels were within normal limits. White blood cell count was also normal, though differential count showed elevated neutrophil percentage (75.8%) and slightly decreased lymphocyte count (18.7%), consistent with a viral infection pattern. No eosinophils were detected. Other values, including creatinine, liver enzymes, total protein, and albumin, remained within reference ranges. These findings supported the clinical impression of a viral etiology and helped exclude significant bacterial superinfection or systemic organ involvement.

Treatment was initiated on the second day after symptom onset with fluconazole (150 mg orally) based on an initial misdiagnosis of oral candidiasis. Due to a lack of clinical improvement, antifungal therapy was discontinued. To manage inflammation and pain in the oral cavity and throat, the patient received prednisolone (20 mg orally) and two doses of intramuscular betamethasone (4 mg). For pain and fever control, a combination of paracetamol with codeine (500 mg/30 mg) and metamizole (500 mg every 6–8 h, as needed) was used. Additionally, ketoprofen (100 mg/day orally) was prescribed for anti-inflammatory support.

To prevent or treat secondary bacterial superinfection, the patient was started on amoxicillin–clavulanic acid (1 g every 12 h orally for 7 days). Local antiseptic care included Hexomedine (0.1% solution oral rinse) administered three times daily. For local ulcer treatment and pain relief, triamcinolone acetonide (0.1% topical, three times daily) was applied to affected mucosal areas.

Despite the lack of antiviral therapy, the patient experienced gradual improvement, with a resolution of fever by day eight and complete recovery within ten days. The fever subsided, and no new oral lesions appeared. By day ten, she was able to speak normally and gradually resumed eating solid foods. Oral ulcerations continued to heal over the following 2–3 weeks with supportive care. No visible scarring or complications were observed. At follow-up, the patient reported a full recovery, apart from experiencing transient fatigue and mild weight loss. No recurrence of symptoms was noted in the subsequent month.

## Discussion

The oral mucosa is frequently involved in various conditions, presenting distinct clinical features. These changes may either be the initial sign of a systemic disease or indicate the activity or progression of the primary condition (1). The lesions reported in this case, which affected the entire oral cavity, cheeks, gums, corners of the mouth, and the posterior pharyngeal wall, could point to viral infections such as herpangina or herpes simplex virus type 1 (HSV-1) infection. Both conditions present with oral ulcerations, fever, and systemic symptoms. The diagnosis was not confirmed through laboratory testing such as PCR, serology, or Tzanck smear, which limits the certainty of the conclusion. This highlights the diagnostic limitations in travel or resource-limited settings.

In the differential diagnosis, other infections should also be considered, such as Epstein–Barr virus (EBV), cytomegalovirus (CMV), and group A streptococcal pharyngitis. EBV and CMV often cause pharyngitis with lymphadenopathy and systemic symptoms, but rarely lead to widespread oral ulcerations. Streptococcal infection may initially present with sore throat, fever, and cervical lymphadenopathy but typically lacks mucosal ulcerations. In this case, the diffuse painful oral ulcers and lack of tonsillar exudate made these diagnoses less likely.

Herpangina is an acute upper respiratory infection, manifesting abruptly with fever, sore throat, and painful herpetic lesions localized to the oral cavity and posterior pharyngeal wall (2). Herpangina is caused by enteroviruses (EVs), including mainly Coxsackievirus (CV) A2, 4, 5, 6, 8, 10, 16 and EV-A71, rarely, CV-B1, 2, 3, 4, and 5 (2). Echovirus 3, 6, 9, 16, 17, 25, and 30 can also cause this disease (3). The virus spreads via the gastrointestinal tract (fecal-oral route) and respiratory system or by contact with oral and nasal fluids. Although herpangina mainly affects preschool children aged 1 to 6, people in all age groups can develop an infection (4, 5). In this case, herpangina was initially misdiagnosed as oral candidiasis. However, white plaques in candidiasis, scraped off, but these lesions were not. These unremovable whitish lesions with a red ring, initially at the base of the tongue, after 2–3 days transformed into small and multiple ulcers. At first, typical for herpangina, acute and painful symptoms were limited to the posterior part of the oral cavity (including the palatine arch, soft palate, uvula, and tonsils), later they began to involve the entire oral cavity (4, 6). Acute gingivostomatitis and systemic symptoms may also suggest HSV infection.

Herpes simplex virus type 1 (HSV-1) can present similarly to herpangina by oral ulcers, fever, and systemic symptoms. Although primary HSV-1 infection usually occurs during the first few years of life, in this case may be plausible as the patient has never had cold sores before. A primary HSV-1 infection in adults can be asymptomatic or present as acute, painful herpetic gingivostomatitis with lymphadenopathy and high fever (38.3 to 40°C). Recurrent HSV infections may be milder and localized. HSV more frequently than enteroviruses causes systemic symptoms such as fever, malaise, and myalgia, especially during primary infection. In contrast, herpangina typically causes mild systemic symptoms with prominent pharyngeal discomfort. Rapid development of orofacial symptoms with generalized oral discomfort and widespread oral ulcers (on the palate, tongue, gums, inside of the cheeks, and in the throat) suggested an HSV infection. The occurrence of severe primary infection is estimated at up to 10% of cases. Regardless of the severity of the primary infection, typically, all signs and symptoms resolve within 10–14 days. In this case, the symptoms and their duration seem to align with the course of primary HSV-1 infection as the patient fully recovered after 10 days (7–9).

HSV may cause more severe and atypical symptoms like herpetic esophagitis or encephalitis in immunocompromised individuals with impaired T-cell immunity (6). In this patient, HIV infection was excluded. Nevertheless, the unusually severe progression of symptoms of acute illness in this patient justifies consideration of another potential underlying immunodeficiency. It underscores the need for further point-of-care diagnostic tools in this context.

Moreover, it should be taken into account that the patient participated in the Brazilian Carnival, which attracts millions of visitors from all over the world. During the clinical interview, the patient reported sharing drinks and having physical contact with multiple individuals during the carnival. These behaviors represent common transmission routes for both HSV and enteroviruses. EVs are transmitted through contact with oral and nasal secretions, fluid from the herpes on skin or mucosa, contaminated hands and objects, etc. (10). This increases the risk of transmission of the virus through direct contact, sharing drinks, and crowded places. Sleep deprivation, alcohol consumption, and poor nutrition can weaken immunity, leading to higher susceptibility to HSV or Coxsackievirus. Moreover, limited hand hygiene and exposure to tropical heat can increase the likelihood of viral infections. Additionally, visitors from different continents may lack immunity to local viral strains, making them more susceptible to infection, as in this case (11).

Due to a lack of routine laboratory tests, most cases of viral infection with oral symptoms are diagnosed clinically. In this case, results of complete blood count (CBC) solely indicated a viral rather than bacterial infection. The diagnosis of herpangina is primarily symptomatic, guided by the pattern of oral lesions and typical symptoms, with PCR testing reserved for atypical or severe cases. Similarly, the diagnosis of HSV infection based on serologic or PCR tests are underutilized in routine clinical practice (12, 13).

Accurate differentiation between HSV infection and herpangina is crucial for effective management, while herpangina resolves spontaneously, HSV requires antiviral treatment (acyclovir, valacyclovir), especially in primary infection. In severe cases (especially in immunocompromised patients), missing early antiviral treatment increases the risk of complications like herpetic esophagitis or encephalitis. In this case of severe stomatitis in an adult was probably caused by primary HSV-1 infection and required antiviral treatment rather than a combined corticosteroid approach. A high dose of corticosteroids administered both topically and orally may have been one of the factors contributing to the unusually extensive course of HSV primary infection. Corticosteroids, while sometimes used to alleviate inflammation, may exacerbate HSV infection by promoting viral replication.

The above-mentioned viral infections are generally considered as trivial and not life-threatening, and as such they are not effectively diagnosed and treated just symptomatically. This case shows however that sometimes they can have a serious clinical course and are associated with pronounced suffering and complications for the patient. They also have a high potential to cause a major outbreak. Until targeted antiviral drugs are developed here, it is worth considering implementing rapid point-of-care diagnostic tools, like rapid COVID/influenza/RSV tests, to guide immediate therapeutic and anti-epidemic actions.

## Conclusion

This case highlights the clinical relevance of viral stomatitis in adults, particularly in young international travelers exposed to high-risk environments such as large public festivals. The atypical presentation and severity of symptoms demonstrate the diagnostic challenges clinicians face when virological testing is not readily available. This underscores the need for rapid point-of-care diagnostic tools, which could facilitate timely differentiation between HSV and enteroviral infections and guide appropriate antiviral therapy.

Importantly, this case also illustrates the potential risks associated with empirical use of systemic corticosteroids without a confirmed diagnosis, especially when HSV infection cannot be excluded. Corticosteroids may worsen viral replication and prolong symptom duration in primary HSV-1 infection.

Finally, there is a need to raise awareness among clinicians regarding the significance of viral oral infections in adults—conditions often perceived as benign or pediatric-only. In reality, they may lead to substantial morbidity, especially in naive hosts exposed to new viral strains during international travel.

## Data Availability

The raw data supporting the conclusions of this article will be made available by the authors, without undue reservation.
